# Blau Syndrome: Challenging Molecular Genetic Diagnostics of Autoinflammatory Disease

**DOI:** 10.3390/genes15060799

**Published:** 2024-06-18

**Authors:** Michaela Brichova, Aneta Klimova, Jarmila Heissigerova, Petra Svozilkova, Manuela Vaneckova, Pavla Dolezalova, Dana Nemcova, Marcela Michalickova, Jana Jedlickova, Lubica Dudakova, Petra Liskova

**Affiliations:** 1 Department of Ophthalmology, First Faculty of Medicine, Charles University and General University Hospital in Prague, U Nemocnice 2, 128 08 Prague, Czech Republic; michaela.brichova@vfn.cz (M.B.); jarmila.heissigerova@vfn.cz (J.H.); petra.svozilkova@lf1.cuni.cz (P.S.); marcela.michalickova@vfn.cz (M.M.); 2 Department of Radiology, First Faculty of Medicine, Charles University and General University Hospital in Prague, Katerinska 30, 128 21 Prague, Czech Republic; manuela.vaneckova@vfn.cz; 3 Department of Paediatrics and Inherited Metabolic Disorders, First Faculty of Medicine, Charles University and General University Hospital in Prague, Ke Karlovu 2, 128 08 Prague, Czech Republic; pavla.dolezalova@vfn.cz (P.D.); dana.nemcova@vfn.cz (D.N.); jana.moravikova@seznam.cz (J.J.); lubica.dudakova@lf1.cuni.cz (L.D.)

**Keywords:** Blau syndrome, early onset sarcoidosis, uveitis, *NOD2*, autoinflammation, neurosarcoidosis

## Abstract

The aim of this study was to describe the clinical and molecular genetic findings in seven individuals from three unrelated families with Blau syndrome. A complex ophthalmic and general health examination including diagnostic imaging was performed. The *NOD2* mutational hot spot located in exon 4 was Sanger sequenced in all three probands. Two individuals also underwent autoinflammatory disorder gene panel screening, and in one subject, exome sequencing was performed. Blau syndrome presenting as uveitis, skin rush or arthritis was diagnosed in four cases from three families. In two individuals from one family, only camptodactyly was noted, while another member had camptodactyly in combination with non-active uveitis and angioid streaks. One proband developed two attacks of meningoencephalitis attributed to presumed neurosarcoidosis, which is a rare finding in Blau syndrome. The probands from families 1 and 2 carried pathogenic variants in *NOD2* (NM_022162.3): c.1001G>A p.(Arg334Gln) and c.1000C>T p.(Arg334Trp), respectively. In family 3, two variants of unknown significance in a heterozygous state were found: c.1412G>T p.(Arg471Leu) in *NOD2* and c.928C>T p.(Arg310*) in *NLRC4* (NM_001199139.1). In conclusion, Blau syndrome is a phenotypically highly variable, and there is a need to raise awareness about all clinical manifestations, including neurosarcoidosis. Variants of unknown significance pose a significant challenge regarding their contribution to etiopathogenesis of autoinflammatory diseases.

## 1. Introduction

Systemic autoinflammatory diseases (SAIDs) are a growing group of disorders caused by a dysregulation of the innate immune system [1]. In most cases, SAIDs have a strong genetic background with pathogenic variants in single genes [2]. To date, 25 genes causing SAIDs have been identified (PanelApp, Autoinflammatory disorders (Version 1.11)) [3]. Nevertheless, 40–60% of patients with phenotypes typical for SAIDs do not obtain distinct diagnoses [2].

Blau syndrome (BS, OMIM #186580), first described by Edward Blau in 1985, is a rare autoinflammatory autosomal dominant disease manifesting as a clinical triad of granulomatous dermatitis, symmetric arthritis and recurrent uveitis [4].

The most frequent symptom reported by 70–96% of patients is joint discomfort, namely redness, swelling, warmth and tenderness [5,6]. Joint manifestations usually present as symmetric, non-erosive polyarthritis and tenosynovitis, involving the wrists, small joints of the hands and feet, ankles and rarely elbows [6,7]. Approximately 60% of patients with BS have camptodactyly, a painless contracture of the proximal interphalangeal joints [6,8,9]. The severity of camptodactyly varies, in some cases being limited to the involvement of only one finger [10]. Generalized skin rash is not accompanied by subjective symptoms [11]. The articular and cutaneous phenotypes manifest early, specifically before 3–4 years of age [6,7,12].

Ocular signs usually start later. The median age at diagnosis is 5 years [13]. The hallmark finding in BS is chronic bilateral uveitis, but conjunctival granulomas, corneal opacities, band keratopathy and optic nerve involvement have also been described [14,15,16]. Uveitis usually presents as granulomatous anterior uveitis or posterior uveitis with multifocal chorioretinal lesions and peripapillary granulomas, but panuveitis is also reported often. The inflammation often persists and impairs visual acuity despite treatment. More than 25% of cases suffer from moderate-to-severe visual loss [16]. Approximately 10–20% of patients do not have ocular involvement in documenting the variability of BS [17,18].

International Blau syndrome registry and rising number of case series reports revealed additional organ manifestations in about one third of the cases [6]. Its features include fever, sialoadenitis, lymphadenopathy, erythema nodosum, leukocytoclastic vasculitis, cutaneous ulcers, transient neuropathies, granulomatous glomerular and interstitial nephritis, interstitial lung disease, arterial hypertension, pericarditis, pulmonary embolism and hepatic and splenic granulomas.

Neurologic findings in BS are described in the literature in several case reports. For example, there is a report of a 36 year-old female with obstructive hydrocephalus due to granulomatous paraventricular lesions [19] and a case with generalized tonic seizures and normal brain magnetic resonance imaging (MRI) [20]. Jabs et al. described in 1985 [21] a family with clinical symptoms consistent with BS and corticosteroid-responsive bilateral neurosensory hearing loss in the proband, as well as transient sixth cranial nerve palsy in their brother. The diagnosis of neurosarcoidosis is based on MRI, cerebrospinal fluid (CSF), electromyography (EMG) or nerve conduction studies documenting findings typical of granulomatous inflammation [22,23,24]. As those methods are nonspecific, tissue biopsy is still considered to be the gold standard, but as it is an invasive procedure, and when considering the risk and benefit, especially in children, it is usually not performed.

In 2001, variants in the *NOD2* gene (OMIM *605956), which encodes nucleotide-binding oligomerization domain protein 2, were identified as causing BS [25]. Two known *NOD2* pathogenic variants, p.(Arg334Trp) and p.(Arg334Gln) (located in exon 4) are responsible for the majority of BS cases, and thus the 334 residue is considered to be the mutational hot spot [7].

Pseudoxanthoma elasticum (PXE, OMIM #264800) is a rare autosomal recessive disease in which dystrophic calcification (i.e., the abnormal accumulation of calcium/phosphate complexes) leads to cutaneous, ocular, cardiovascular and other manifestations. The first clinical sign of PXE is almost always small yellow papules on the neck and in flexural areas that coalesce over time, while the skin becomes loose and wrinkled. Dystrophic calcification of the retinal Bruch membrane appears as angioid streaks. Lesions in small- and medium-sized artery walls may result in intermittent claudication and peripheral artery disease. Cardiac complications (myocardial infarction and angina pectoris) and ischemic strokes have also rarely been observed [26]. PXE is caused by mutations in the *ABCC6* gene (OMIM *603234), which encodes the protein called multidrug resistance-associated protein 6 (MRP6), also known as the ABCC6 protein [27].

In this study, we report the clinical findings and results of *NOD2* screening in three families with the occurrence of BS. In addition to the typical BS phenotype, we also document rare neurological signs of the disease spectrum and a dual diagnosis of PXE and BS.

## 2. Materials and Methods

Participants were enrolled at the Department of Ophthalmology of the First Faculty of Medicine at Charles University and General University Hospital in Prague. The study adhered to the tenets of the Declaration of Helsinki and was approved by the institutional review boards of General University Hospital in Prague (reference number 34/19). All subjects or their legal guardians provided informed consent prior to inclusion in the study.

Genomic DNA was isolated from peripheral blood using a Gentra Puregene™ Blood Kit (Qiagen, Hilden, Germany) or saliva samples using an Oragene Saliva Collection and DNA extraction kit (Genotek, Ottawa, Canada) according to the manufacturer’s instructions.

In all 3 probands, exon 4 of the *NOD2* gene was PCR amplified using previously published primers [28] and bidirectionally analyzed through conventional Sanger sequencing on a 3500 Series Genetic Analyzer (Applied Biosystems, Foster City, California, USA). NM_022162.3 was taken as the reference sequence. In the proband from family 3 (individual III:1, Figure 1) and their sister (III:2, Figure 1), targeted next-generation sequencing using an autoimmune panel containing 89 genes was performed (Supplementary Materials).

Next, exome sequencing was performed in the mother of proband 3 using a SureSelect Human All Exon V6 Kit (Agilent, Santa Clara, California, USA) and sequenced on a NovaSeq 6000 instrument (Illumina, San Diego, California, USA) with 150 bp paired-end reads. The sequence reads were analyzed via the Franklin platform as previously described [29]. Sanger sequencing was used for segregation analysis within the families.

Variants with a minor allele frequency <0.005 as mined from the Genome Aggregation Database (gnomAD v.4.0.0) [30] in genes associated with autoinflammatory disorders (PanelApp, version 1.1) and the arthrogryposis panel (version 5.13, which includes all known genes causing camptodactyly) were filtered [3]. The pathogenicity of each variant was evaluated according to the recommendations of the American College of Medical Genetics and Genomics and the Association for Molecular Pathology (ACMG-AMP) [31]. The final variant scoring for one of the five categories (pathogenic, likely pathogenic, variant of unknown significance (VUS), likely benign or benign) was performed using the Franklin platform (https://franklin.genoox.com, accessed 8 May 2024).

The ophthalmic examination included intraocular pressure and Snellen best corrected visual acuity (BCVA) measurements converted to decimal values, as well as slit lamp examination of the anterior segment and fundus in pupil dilation. Wide-field fundus photographs were taken using a Clarus 700 and FF 450 plus IR (Carl Zeiss Meditec AG, Jena, Germany). Spectral domain optical coherence tomography (SD-OCT) was performed using Spectralis (Heidelberg Engineering GmbH, Heidelberg, Germany).

The general medical work-up encompassed a metabolic panel (blood urea nitrogen, creatinine, fasting glucose and liver function tests), blood cell count and C-reactive protein analysis. The rheumatoid factor, HLA B27 antigen, antinuclear antibodies, antineutrophil cytoplasmic antibodies and serum levels of chitotriosidase were analyzed, and the angiotensin-converting enzyme was measured in patients examined by a rheumatologist. Other complementary investigations undertaken comprised blood pressure measurement, abdominal ultrasonography (US), chest X-rays, echocardiography and brain MRI, which was carried out with a 3 Tesla (T) MRI scanner (MAGNETOM Skyra, Siemens Healthcare, Erlangen, Germany). The protocol comprised 3D T1 magnetization-prepared rapid acquisition with gradient echo (MPRAGE), 3D fluid attenuated inversion recovery (FLAIR), 2D T2-weighted images (T2WIs) in transversal and coronal cuts and diffusion-weighted images (DWIs) in transversal cuts.

## 3. Results

Seven individuals (six females, one male; 14–66 years old; mean age: 30 years) from three families with clinical signs of BS were included in the study.

The proband from family 1 (II:1, Figure 1) developed maculopapular exanthema of the trunk, extremities and face at the age of 3 months (Figure 2A). Granulomatous inflammation was confirmed by skin biopsy. Symmetrical polyarthritis of large and small joints (55 in total) appeared at the age of 3 years, leading to a referral to a tertiary pediatric care center as a juvenile idiopathic arthritis (JIA) suspect.

Visual acuity at 3 years was 0.5 in the right eye (RE) and 0.3 in the left eye (LE), and the intraocular pressure was within the normal limits. Upon ophthalmic examination, there were bilateral subepithelial corneal opacities considered to be a result of resorption of previous corneal granulomas (Figure 2C,D). Relapses of chronic anterior uveitis were observed up to 7 years, and until 12 years, the subject gradually developed peripapillary, choroidal and retinal granulomas classified as chronic panuveitis (Figure 2B). Despite local anti-inflammatory treatment and continuous systemic immunomodulatory therapy (corticosteroids, methotrexate and mycophenolate mofetil) and biologics (adalimumab, infliximab, anakinra, abatacept and tocilizumab), there were repeated episodes of bilateral panuveitis. During anti-IL1 treatment at 11 years, an increase in uveitis activity was observed, together with inflammation of the parotid and lacrimal gland and an episode of meningoencephalitis. Therefore, after 4 months, the anti-IL1 treatment was replaced by anti-IL6 treatment. Between 7 and 11 years, the patient underwent bilateral cataract surgery and five anti-glaucoma procedures (cyclophotocoagulation and trabeculectomy bilaterally, in addition to deep sclerectomy with a drainage system in the RE).

Acute episodes of meningoencephalitis presented at 5 years with seizures, vomiting, fever, right hemiparesis and confusion and at 11 years with headaches, confusion, and vomiting. A brain MRI was performed and revealed numerous small foci in the white matter, and cerebrospinal fluid analysis showed pleocytosis (Figure S1A). The findings were considered by a neurologist as possible neurosarcoidosis. One month after the second episode of meningoencephalitis, enlargement of the parotid (Figure 2E) and lacrimal glands followed (Figure 2F), which was considered to be granulomatous inflammation due to the granular appearance on US imaging.

The enzyme chitotriosidase (normal levels: 4.4–89.0 nmol/h/mL) is considered to be a reliable biomarker for monitoring inflammatory activity [32,33,34]. In the proband from family 1, its serum levels increased from a baseline of 43–63 nmol/h/mL to 99 nmol/h/mL during the period of parotid and lacrimal gland enlargement.

At the last follow-up (age: 14 years), the patient had uveitis in remission being treated systemically with the Janus kinase inhibitor baricitinib, which was initiated at 12 years, low doses of prednisone and mycophenolate mofetil. Her BCVA was 0.5 in the RE and 0.4 in the LE. Bilateral visual field defects were attributed to glaucomatous optic nerve damage.

Arthritis and dermatitis were in remission since 4 years of age. The arthritis subsided after 9 months, as did the dermatitis after 1 month from the start of systemic immunosuppression.

The proband from family 2 (II:2, Figure 1) suffered from joint problems since the age of 8 years, when she developed symmetrical tenosynovitis of the wrists (Figure 3C) and ankles. The tenosynovitis became chronic, with activity in the US but without erosive arthritis in X-rays. She was followed for relapsing activity in the anterior chamber since 11 years of age. When aged 24 years, ophthalmic examination revealed bilateral panuveitis with corneal opacities, posterior synechiae, iris granulomas, vitreous opacities and several chorioretinal, atrophic, partially pigmented lesions mainly in the inferior half of the retina (Figure 3A,B,D–F). The BCVA was 1.0. The relapse of activity in the anterior chamber and vitreous along with symmetrical tenosynovitis of the dorsal wrists and anterior ankles despite topical treatment led to the introduction of systemic therapy using adalimumab at 34 years. At the last examination, aged 35, the BCVA remained at 1.0 in both eyes.

Additional examinations including chitotriosidase were within the normal limits. Dermatitis was present at preschool age but was resolved with topical medication and did not recur thereafter.

The proband from family 3 (III:1, Figure 1) was referred at 12 years old with a one-year history of panuveitis. The BCVA was 1.0 in both eyes. Conjunctival granulomas, corneal opacities, posterior synechiae and a few chorioretinal, atrophic, partially pigmented circular lesions in the periphery were found (Figure 4A–C). She had flexion deformities in the elbows and ankles and camptodactyly of the fourth finger bilaterally (Figures S2A and S3A). She underwent surgery of the right hallux valgus and the left ankle for congenital equinus contracture at 1 and 8 years, respectively. Since 13 years of age, she has been regularly checked by a rheumatologist, and active arthritis has not been detected. However, its development could have been mitigated by continuous systemic anti-inflammatory treatment. A brain MRI revealed three small foci in the white matter (Figure S1B).

At preschool age, she suffered from a non-itching generalized severe skin rash treated as atopic dermatitis, which completely resolved upon topical corticosteroid treatment within 2 years, and no skin problems occurred thereafter. Treatment with methotrexate started at the age of 12 years to manage active uveitis, predominantly in the anterior segment. When discontinued due to inactivity 4 years later, the anterior uveitis relapsed, and treatment was resumed. For the last ophthalmic examination at 17 years old, the uveitis was in remission. The BCVA remained at 1.0 bilaterally.

The monozygotic triplet sister (III:2, Figure 1) had joint problems from 6 years of age, when she developed tenosynovitis in both ankles, which was originally diagnosed as JIA. From this indication, she has been treated with methotrexate since 9 years of age. She also had camptodactyly in fingers three and four bilaterally as well as toe five (Figures S2B and S3B). She underwent several surgeries on the bilateral congenital pes equinovarus between 1 and 8 years and the left hallux valgus at 13 years.

In her preschool years, she had a mild skin rash on the trunk and extremities that did not itch and resolved after topical corticosteroids. Ophthalmic examination revealed conjunctival granulomas, corneal opacities and asymptomatic posterior uveitis characterized by a few chorioretinal, partially pigmented lesions in the periphery, predominantly in the inferior region. At the last follow-up (aged 17 years), the BCVA was 1.0, and the uveitis was inactive in both eyes.

**Figure 1:**
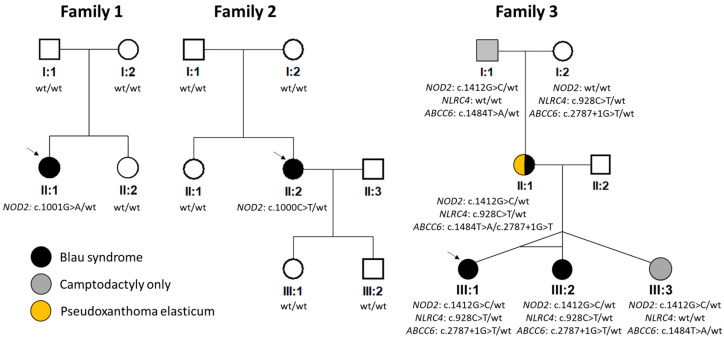
Pedigrees and results of segregation analysis. NM_022162.3 (*NOD2*), NM_001199139.1 (*NLRC4*) and NM_001171.6 (*ABCC6*) were taken as reference sequences. Wt = wild type. Arrows indicate probands.

**Figure 2:**
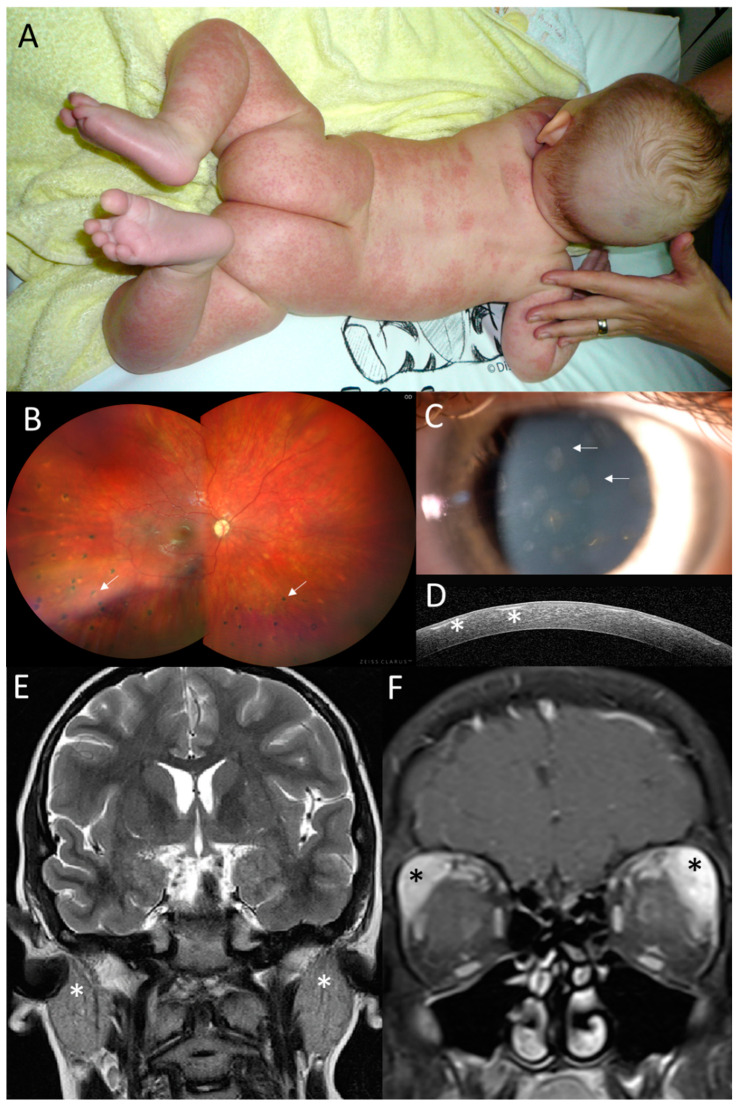
Clinical findings in the proband from family 1. (**A**) Granulomatous maculopapular rash on trunk and extremities at 3 months. (**B**) Pale optic disc and multifocal chorioretinal lesions in the right eye (arrows) located predominantly in the inferior half at 12 years. (**C**) Anterior segment photograph of subepithelial corneal opacities in the right eye (arrows) and (**D**) their visualization on SD-OCT corneal section (asterisks) at 14 years. (**E**) Coronal T2WI and enlargement of parotid glands (asterisks) at 11 years of age. (**F**) Coronal T1WI + FatSat+ Gd and enlargement of infiltration lacrimal glands, which is more prominent on the left side, at 11 years of age (asterisks).

**Figure 3:**
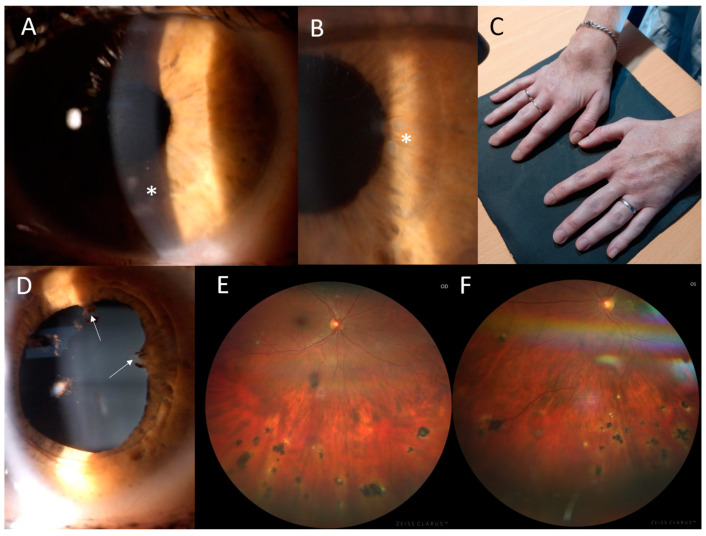
Clinical findings in the proband from family 2. (**A**) Corneal opacities in the left eye, located mostly in the inferior half (asterisk). (**B**) Multiple iris granulomas in the right eye (asterisk). (**C**) Symmetrical dorsal wrist edema. (**D**) Posterior iris synechiae in the right eye (arrows). (**E**) Chorioretinal partially pigmented atrophic lesions located in the inferior half of the retina in the right eye and (**F**) in the left eye.

The dizygotic triplet sister (Figure 1, individual III:3), examined for the first time when aged 10 years and 17 years at the last follow-up visit, had no joint problems or uveitis. She had camptodactyly of fingers four and five (Figure S2C). She underwent bilateral hallux valgus surgery at 13 years.

**Figure 4:**
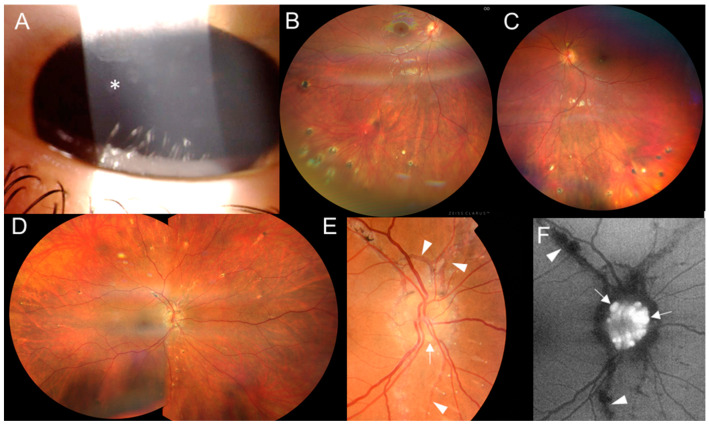
Clinical findings in two individuals from family 3. Proband III:1’s (**A**) corneal opacities (asterisk) in the right eye and (**B**) inactive chorioretinal lesions in the inferior periphery in the right eye and (**C**) left eye. Individual II:1’s (**D**) inactive chorioretinal lesions scattered in the periphery circumferentially in the right eye and (**E**) optic nerve drusen in the right eye (arrows), with angioid streaks (arrowheads) in the color fundus photo and (**F**) in the fundus autofluorescence photo.

The mother of the triplet sisters (Figure 1, individual II:1), examined at 43 years, had bilateral signs of inactive posterior uveitis with peripheral, chorioretinal, partially pigmented lesions and optic nerve drusen (Figure 4D–F). In addition, a typical manifestation of PXE, angioid streaks, was found (Figure 4E,F), along with papules located on the lateral side of the neck. She denied the presence of skin rush in childhood. Her BCVA was 1.0 in both eyes. Systemic evaluation revealed marked obesity (body mass index: 55) and symmetrical lymphedema in both legs. She has been treated for arterial hypertension and given substitution therapy for hypothyroidism. She also had marked camptodactyly of fingers 3–5 (Figure S2D) but never suffered from arthritis. Other examinations showed two small foci in a brain MRI (Figure S1C), diffuse liver hyperechogenicity, cholecystolithiasis and splenomegaly in an abdominal US.

The 66 year-old grandfather of the proband from family 3 (I:1; Figure 1) never reported skin or joint problems, though he has been treated for type 2 diabetes mellitus. He had mild camptodactyly of fingers four and five (Figure S2E). There were no signs of uveitis upon ocular examination. Supplementary examinations showed three nonspecific small foci in the white matter of a brain MRI (Figure S1D).

The clinical findings of all individuals investigated are summarized in Table 1.

Molecular genetic analysis of the *NOD2* gene revealed in families 1 and 2 previously reported heterozygous pathogenic variants c.1001G>A p.(Arg334Gln) and c.1000C>T p.(Arg334Trp), respectively [12,25]. As parents of both probands did not carry the change, it likely appeared de novo (Figure 1).

In family 3, the c.1412G>C p.(Arg471Pro) in *NOD2* was found in the heterozygous state in all five family members (Figure 1), of which only one had a classical triad of BS including uveitis, and two had uveitis, one of whom had documented skin rush, while two exhibited camptodactyly only. This variant has not been reported in the literature, and it is not present in the gnomAD v.4.0.0 dataset. However, the meta-predictor REVEL score for this variant was 0.14 (i.e., in the range of moderately benign). Based on the ACMG-AMP criteria, the c.1412G>C in *NOD2* was classified as a VUS.

To exclude other possible causal variants, screening using a panel containing genes known to be associated with autoimmune or autoinflammatory disorders was performed in the proband and her monozygotic twin sister. Except for c.1412G>C in *NOD2*, another heterozygous variant classified as a VUS was identified: c.928C>T p.(Arg310*) in *NLRC4* (NM_001199139.1). Segregation analysis showed that this change was inherited from an unaffected maternal grandmother and hence unlikely to be disease-causing (Figure 1). In addition, the *NLRC4* gene has a pLI of zero, suggesting that loss-of-function variants are tolerated [35].

Exome sequencing in the mother of the proband from family 3 (II:1, Figure 1) was also performed to identify the molecular genetic cause of PXE and exclude the presence of rare variants in other genes known to be associated with camptodactyly. Two previously reported pathogenic variants in *ABCC6* (reference sequence: NM_001171.6) were found: c.2787+1G>T p.? and c.1484T>A p.(Leu495His) [36]. Subsequent segregation analysis (Figure 1) confirmed their trans configuration. Identification of these two mutations explained the presence of angioid streaks and skin papules as signs of PXE. No other rare, possibly pathogenic variants in genes associated with camptodactyly were identified.

## 4. Discussion

Herein, we reported two cases with BS confirmed by the identification of previously reported pathogenic variants—c.1001G>A and c.1000C>T—and one family with two novel heterozygous changes—c.1412G>C in *NOD2* and c.928C>T—in *NLRC4*, classified as a VUS. The presumed mechanism of BS-causing mutations is faulty canonical signaling and reduced IRF4-mediated cross-regulation [37].

The de novo occurrence of BS has been reported in 64% of cases [17]. In our study, it was present in two out of the three families. It needs to be noted, however, that paternity and maternity testing were not performed.

The clinical manifestation and inflammatory activity of BS signs can be quite variable, as was also documented in this study. This is not surprising, given the fact that BS shows incomplete penetrance, which was reported to be 9–23% in various cohorts, and camptodactyly may be the only sign of BS [18,38].

Both individuals with known *NOD2* pathogenic variants developed a classical BS triad (granulomatous recurrent uveitis, dermatitis and arthritis). The clinical findings in family 3 were more variable; the classical triad was present in one individual, one member had signs of recurrent uveitis and skin rash, and one had only inactive uveitis. The limitation of the current study is that to prove the causality of c.1412G>C in *NOD2*, more BS patients carrying the same variant need to be identified.

All five heterozygous carriers of c.1412G>C in *NOD2* had camptodactyly. Interestingly, uveitis was only present in members who also harbored c.928C>T in *NLRC4*. The possible contribution of this variant to the disease’s pathogenesis remains to be elucidated. The *NLRC4* gene (OMIM *606831) encodes a cytoplasmic (nucleotide-binding and oligomerization domain)-like receptor that can trigger inflammasome formation in response to bacterial flagellin, an immunodominant antigen in the intestine [39]. While *NLRC4*’s gain-of-function mutations have been associated with early-onset recurrent fever, recurrent macrophagic activation syndrome and enterocolitis [40], loss-of-function variants are predicted to be tolerated.

Upon ocular examination, we observed conjunctival granulomas in three cases. Four individuals had corneal opacities, while multifocal chorioretinal lesions were observed in five subjects. During follow-up, active uveitis requiring systemic treatment was present in three individuals. Joint swelling was initially present in three subjects, appearing in one as polyarthritis and in two as tenosynovitis of the ankles or wrists. Camptodactyly, reported in approximately 60% of BS cases, was found only in family 3 to variable degrees in the hands and in the feet. Among all five members investigated, hallux valgus deformity requiring surgery in the first or second decade of life was noted in three of them. As this feature has not been associated with BS in the literature, this is likely to be a coincidence. We also considered that camptodactyly could be an independent trait or sign of a different disease [10,41]. For this reason, one individual underwent exome sequencing, with a bioinformatical assessment of genes reported to be associated not only with autoinflammatory but also connective tissue disorders. No other causal variants were found in the candidates.

For the brain MRI, four subjects had hyperintense, nonspecific foci in their white matter, which may have led to a suspicion of neurosarcoidosis (Figure S1), as documented in other observations [42]. All but one subject were neurogically asymptomatic. One patient had two episodes of meningoencephalitis and inflammation of the parotid and lacrimal glands, which subsided rapidly after a high dose of methyprednisolon.

Children with BS are often misdiagnosed as having atopic dermatitis or JIA. In our study, three individuals were observed for atopic dermatitis at preschool age, and two subjects were initially diagnosed with JIA. For differential diagnosis, it is important to emphasize that JIA-associated uveitis is mostly confined to the anterior segment [43,44], while BS can result in prominent fundus inflammatory lesions.

Due to the rarity and variability of BS, diagnostic delay is common. In our study, this ranged from 2 to 23 years (mean: 9 years). It is also likely that many BS cases remain undiagnosed due to nonspecific granulomatous clinical manifestation, good therapeutic response and low levels of awareness and genetic testing.

## 5. Conclusions

BS is an early-onset, multiorgan, autosomal dominant disease. Dermatitis, followed by arthritis or tenosynovitis and uveitis, which can be sight-threatening and requires systemic treatment, is present in approximately 50% of cases, while extra-triad manifestations occur in about one third of cases. Clinicians involved in the care of BS patients should be aware of the full disease spectrum.

## Figures and Tables

**Table 1:** Summary of clinical findings in 7 individuals from 3 families with Blau syndrome.

Family/Individual	Sex/Age at Last Ophthalmic Examination	Skin Rash/Age at Presentation	Arthritis or Tenosynovitis/Age at Presentation	Corneal Opacities	Ocular Findings/Age at Presentation	Camptodactyly	Other Clinical Finding	Relevant Laboratory Findings	Systemic Treatment for Blau Syndrome	Follow-Up Duration
F1/II:1	F/14 y	3 months	polyarthritis/3 y	Y	active panuveitis/3 y	N	meningoencefalitis (presumed neurosarcoidosis) at age 5 y and 12 y, multifocal foci in white matter, parotitis and dacryoadenitis at 12 on MRI	serum chitotriosidase, 43–99 nmol/h/mL	therapy from 4 y for uveitis, baricitinib at the last follow-up, prednisone, MMF, previously MTX, hydroxychloroquine, anti-IL1, anti-IL6, antiTNFα	13 y
F2/II:2	F/35 y	preschool	tenosynovitis of wrists and ankles/8 y	Y	active panuveitis/11 y	N	N	N	adalimumab from 34 y for uveitis and tenosynovitis	23 y
F3/III:1	F/17 y	preschool	probable polyarthritis/preschool	Y	active panuveitis/12 y	Y	3 small foci in white matter on MRI, bronchial asthma	N	MTX from 12 y for uveitis	5 y
F3/III:2	F/17 y	preschool	tenosynovitis of ankle/6 y	Y	inactive posterior uveitis/NA	Y	cholecystolithiasis	N	MTX from 9 y for tenosynovitis	4 y
F3/III:3	F/17 y	N	N	N	N	Y	N	N	N	NA
F3/II:1	F/44 y	N	N	N	angioid streaks, inactive posterior uveitis/NA	Y	2 small foci in white matter in brain MRI, hepatopathy, hypothyroidism, cholecystolithiasis, splenomegaly, lower extremities lymphedema, hypertension, obesity	TSH, 5.7 mU/l	N	NA
F3/I:1	M/66 y	N	N	N	N	Y	3 small foci in white matter in brain MRI, hepatopathy, diabetes mellitus	N	N	NA

Y = yes; N = none; M = male; F = female; NA = no available data; MTX = methotrexate; MMF = mycophenolate mofetil; y = years, TSH = thyroid-stimulating hormone.

## Data Availability

The data that support the findings of this study are available from the corresponding authors upon reasonable request.

## Supplementary Materials

The following supporting information can be downloaded at https://www.mdpi.com/article/10.3390/genes15060799/s1. List of genes on autoimmune gene panel. Figure S1: Small nonspecific hypersignal foci in the white matter of the brain in FLAIR MRI image. Figure S2: Camptodactyly in five individuals from family 3. Figure S3: Joint deformities in X-ray images for family 3.
