# Supplementary material for: Blau Syndrome: Challenging Molecular Genetic Diagnostics of Autoinflammatory Disease
Source: Genes (Basel). 2024 Jun 18;15(6):799. doi: 10.3390/genes15060799 (PMC11203189; doi:10.3390/genes15060799)
Supplement: Supplementary file 1 [file genes-15-00799-s001.zip › genes-3029362-supplementary.pdf]

List of genes on autoimmune gene panel:
ADAM17, AP3B1, C1QA, C1QB, C1QC, C1R, C2, C3, C4A, C5, C6, C7, C8A, C8B, C9, CARD14, CARD8, CASP10, CASP8, CECR1(ADA2), CFH, CFHR5, CFI, CFP, COL3A1, COL5A1, COL5A2, CTLA4, DNASE2, DOCK8, ELANE, ELN, FAS, FASLG, FOXP3, G6PC3, HAX1, IKBKG, IL10, IL10RA, IL10RB, IL1RN, IL21, IL22, IL36RN, LPIN2, LRBA, LYST, MALT1, MASP2, MBL2, MEFV, MVK, NCF2, NLRC4, NLRP12, NLRP3, NLRP6, NLRP7, NOD2, NRAS, OTULIN, (FAM105B), PLCG2, PLOD1, PRF1, PRG4, PSMA3, PSMB4, PSMB8, PSMB9, PSTPIP1, RAB27A, SEC16A, SERPING1, SH2D1A, SLC29A3, STX11, STXBP2, TMEM173, TNFAIP3, TNFRSF11A, TNFRSF1A, TRAP1, TRNT1, TTC7A, UNC13D, WAS, WDR1, XIAP

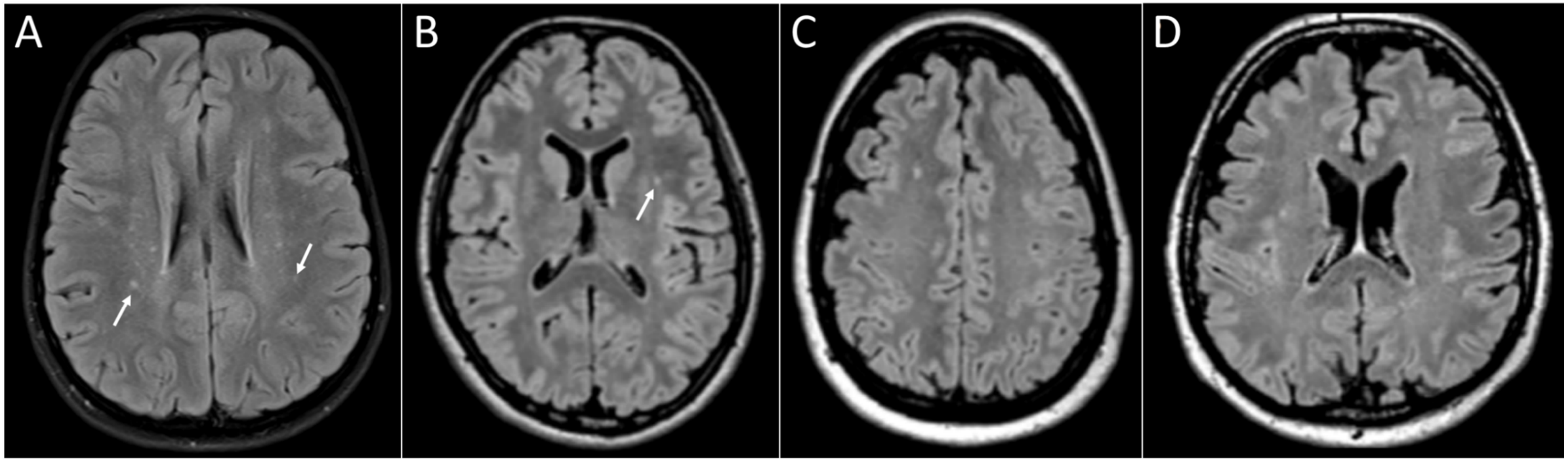

Figure S1. Small nonspecific hypersignal foci in the white matter of the brain on FLAIR MRI image (arrows) in proband from family I (A), proband from family 3 (B), her mother (C) and grandfather (D).

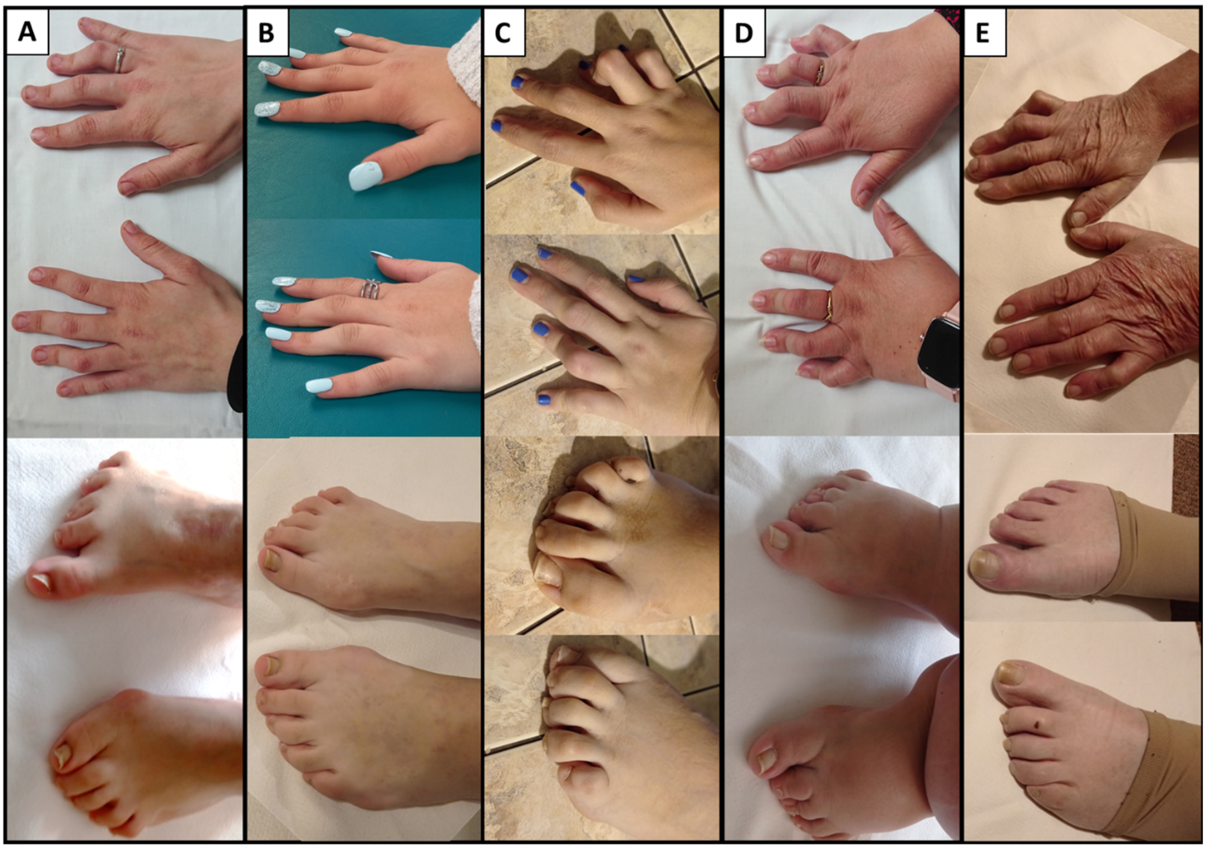

Figure S2. Camptodactyly in five individuals from family 3. Family members A) III:1, B) III:2, C) III:3, D) II:1 and E) I:1.

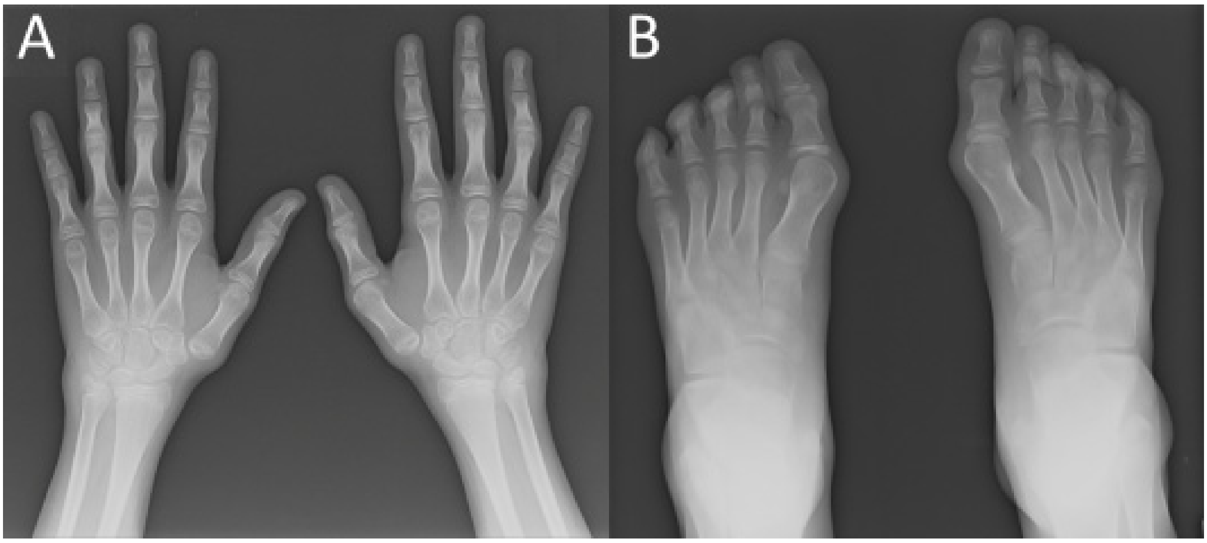

Figure S3. Joint deformities on X-ray images present in family 3. Family member III:1 showing camptodactyly of finger IV in the right hand (A), and family member III:2 showing bilateral joint deformities of toes II-V including hallux valgus before surgery (B).
